# Non-Contact Body Measurement for Qinchuan Cattle with LiDAR Sensor

**DOI:** 10.3390/s18093014

**Published:** 2018-09-09

**Authors:** Lvwen Huang, Shuqin Li, Anqi Zhu, Xinyun Fan, Chenyang Zhang, Hongyan Wang

**Affiliations:** 1 College of Information Engineering, Northwest A&F University, Yangling, Xianyang 712100, China; chenyangcheung@nwsuaf.edu.cn; 2 Key Laboratory of Agricultural Internet of Things, Ministry of Agriculture and Rural Affairs, Yangling, Xianyang 712100, China; 3 School of Information Management, Wuhan University, Wuhan 430072, China; zhuanqi_sim@whu.edu.cn; 4 College of Computer Science, Wuhan University, Wuhan 430072, China; fxy.rebecca@163.com; 5 Western E-commerce Co., Ltd., Yinchuan 750004, China; nxwhy01@126.com

**Keywords:** body dimensions, filtering, clustering, RANSAC, segmentation, ICP matching, reconstruction, FPFH

## Abstract

The body dimension measurement of large animals plays a significant role in quality improvement and genetic breeding, and the non-contact measurements by computer vision-based remote sensing could represent great progress in the case of dangerous stress responses and time-costing manual measurements. This paper presents a novel approach for three-dimensional digital modeling of live adult Qinchuan cattle for body size measurement. On the basis of capturing the original point data series of live cattle by a Light Detection and Ranging (LiDAR) sensor, the conditional, statistical outliers and voxel grid filtering methods are fused to cancel the background and outliers. After the segmentation of *K*-means clustering extraction and the RANdom SAmple Consensus (RANSAC) algorithm, the Fast Point Feature Histogram (FPFH) is put forward to get the cattle data automatically. The cattle surface is reconstructed to get the 3D cattle model using fast Iterative Closest Point (ICP) matching with Bi-directional Random K-D Trees and a Greedy Projection Triangulation (GPT) reconstruction method by which the feature points of cattle silhouettes could be clicked and calculated. Finally, the five body parameters (withers height, chest depth, back height, body length, and waist height) are measured in the field and verified within an accuracy of 2 mm and an error close to 2%. The experimental results show that this approach could be considered as a new feasible method towards the non-contact body measurement for large physique livestock.

## 1. Introduction

The variation in body dimensions of cattle during their growth periods correlates with body weight [1], productivity evaluation [2], selection and breeding [3]. The periodical measurement of body dimensions is used to evaluate the growth response to nutrient supply and health anomalies [4], which is one of the most primary quality evaluation criteria [5]. However, sufficient frequency of body sizes measurement is not easy to accomplish; basically, the body size of adult cattle is measured monthly using scales where the cattle need to be placed in a holding frame which can be stressful for the cattle as well as labor intensive for the farmer. There is therefore a need to automate the body measuring process as well as make it easier to measure body sizes whenever needed. This paper presents a non-contact body dimensions measurement approach for Qinchuan cattle, which is a safe, proximal and convenient method for measuring the body sizes of live cattle, and also can be extended to body measuring applications for other large animals.

### 1.1. Agricultural Applications of LiDAR

A wide region of agricultural applications can be found in the literature with the aim of developing a sensor-based three-dimensional (3D) reconstruction method for contactless measurements. Light Detection and Ranging (LiDAR) technology is generally used for many purposes of remote sensing of realistic 3D image information [6]. The point cloud data (PCD) acquired by a LiDAR sensor can reflect the 3D mapping of research targets or automated environmental monitoring data. Regarding the applications of the Kinect RGB-D camera, it is widely applied and convenient for direct proximal data acquisition of research targets, such as the estimation of fruit sizes of on-tree mangoes [7], 3D measurements of maize plant height [8] and automated behavior recognition of pigs [9]. For the planar scanning applications of SICK LMS series sensors, larger range of view sight data could be obtained, such as 3D plant reconstruction of maize [10], fruit yield mapping of individual trees in almond orchards [11] and leaf area index estimation in vineyards [12]. Related studies have been performed and extended to airborne LiDAR for measuring 3D distributions of plant canopies of landscape [13], post-harvest growth detection [14], diversity distribution of Mediterranean forests [15], leaf area index estimation [16] and some outdoor object reconstructions [17] and so on. These successful studies suggest that proximal or contactless sensing of large animals is possible instead of manual measuring.

Due to the wide applications of LiDAR technology, many processing and analysis methods as well as feature descriptors extraction, segmentation and reconstruction algorithms of PCD were proposed for different purposes [18,19,20]. In [21], a Model Point Feature Histogram (MPFH) has been presented for recognizing surface defects in 3D point clouds, and it has been reported that the rate of recognition of defects was close to 94%. A generative model in a Euclidean ambient space with clusters of different shapes, dimensions, sizes, and densities has been considered theoretically [22]. A cell-based way to identify the outliers and an approximate approach using a bounded Gaussian distribution have been proposed [23]. A Fast Point Feature Histograms (FPFH) with a SAmple Consensus-based Initial Alignment (SAC-IA) algorithm has been presented for 3D point data registration for nonlinear optimization problems [24]. Fitting and registration algorithms have been developed for different occasions and applications, such as 3D-reconstruction from irregularly distributed and noisy point data [25], B-spline surface reconstruction [26] and greedy geometric algorithm [27] and so on. All the processing and analysis methods of PCD have achieved good effects in specific fields, and could provide the references for methods and algorithms suitable for measuring the body sizes of cattle.

### 1.2. Imaging Systems for Body Measuring

The computer vision approach with LiDAR sensors has been widely used for animal body measurements [28]. The body sizes of cattle could be remotely estimated by imaging techniques, and some successful studies with different cameras have been applied for different scientific purposes [29]. Most digital imaging methods may have certain limitations when face by changes in illumination and background, which causes noise that affects the segmentation correctness and may lead to erroneous estimated results [30]. It is a great challenge to capture the body contour information of cattle on the spot because they are animals and always are moving [31]. However, with the specific application extension of LiDAR [32], the availability of 3D digital models of real objects is becoming a new research focus, such as body condition scoring for beef cattle [33], lame walking detection [34,35], backfat thickness estimation for lactating Holstein-Friesian cows [36], body condition scoring between Holstein Friesian cows (Karkendamm) and Fleckvieh (Grub) breeds [37], assessments of rump fat and muscle score in Angus cows and steers [38] and so on. All these achievements provide some effective 3D sensing models to capture the silhouette image despite the mostly similar color of cattle.

Several studies have assessed the feasibility of utilizing digital images or 3D imaging analysis to determine body condition and weight for dairy cows. Four body dimensions of Holstein cows (withers height, hip height, body length, and hip width) have been determined for predicting live weight by a fuzzy rule-based color imaging system [39], which have been deployed four different directions of Cannon cameras after calibration [40]. For continuous 3D body reconstruction of calves, young and adult cows, an automated measuring system with Kinect cameras has been validated for live weight estimation of each cow, and five body dimensions of cows (hip and withers height, hips distance, head size, chest girth) have been measured with different estimation coefficients [41]. Certain methods for live weight measuring for cattle have been achieved and referred to body dimensions, but no clear specific patterns and methods for the standing body dimensions of live cattle could be observed.

For body measuring of other animals, a considerable body of literature has accumulated relating to body measuring for livestock and visual analysis theory. For measuring the body sizes of pigs, researchers have proposed a portable and automatic measuring system equipped with an ASUS Xtion Pro camera to measure three body dimensions of live pigs (body width, hip width, and body height) with average relative errors within 10.30% [42]. With the application of a Kinect sensor, aspects of the body measurement of pigs have been investigated, such as automatic recognition of aggressive behavior [9], real-time monitoring for touching-pigs [43], live weight determination of pigs from measured dimensions [3,44], normal walking patterns assessment [28] and so on. For remote measuring with 3D PCD, a series of processing techniques or methods can be employed, such as parameter calibration, Euclidian clustering, RANdom SAmple Consensus (RANSAC) segmentation, viewpoint feature histogram (VFH) extraction, PCD registration, grid reconstruction as well as body measurement.

For the body sizes measurements of sheep, in reference [45], a low-cost dual web-camera has been used to observe the weights of live Alpagota sheep in terms of three body dimensions (withers height and chest depth and body length) within a mean error of 5%. A visual measuring method with industrial cameras has been presented to measure seven body dimensions (back height, rump height, body length, chest depth, chest width, abdominal width and rump width) of small-tailed Han sheep, and the on the spot experimentation results of ten sheep have shown the over 90% of the errors of are within 3% [46]. Similarly, the color imaging analysis for body measuring have been deployed for pigs [47,48,49], and these studies have been isolated or structure light deployment in case of poor light limitations and more complex noises.

### 1.3. Main Purposes

From the non-contact visual measuring methods proposed for cows, pigs, or sheep, the remote sensing system of body sizes with LiDAR sensors offers one large advantage with its lack of limitations caused by poor lighting. 3D point cloud imaging systems are ready-to-use and easily transportable, and have been widely employed to acquire different shape of large cows or cattle. Qinchuan cattle is most excellent beef breed in China [50]. The average withers height of an adult Qinchuan cow is about 132 cm, and its average body weight is 420 kg, whilst the average withers height of an adult Qinchuan bull is about 148 cm and its average body weight is 820 kg. To produce non-contact measurements of the body dimensions for a large physique breed with LiDAR sensors, there exist two different key issues, which involve how to find a general filtering solution to obtain the clear and complete contour of the cattle, and how to calibrate the LiDAR sensor to acquire precise measurement data. For the first key problem, different filtering and threshold selection methods could be tested and analyzed in field experiments. The LiDAR data processing and analysis in our work [6] and other techniques [51] provide the references for new trials. For the second key issue, the measuring calibration or surface model correction in our work [52] has been validated and achieved an accuracy of 2%. For this approach, the original contributions can be summarized as follows:

- New filter fusion and clustering segmentation methods are presented, where the filter fusion can effectively remove the uneven distribution of PCD, multiple noises and many outliers, and the clustering segmentation can accurately extract the spatial position, geometry shape and proximity distance for the cattle.
- The feature extraction, matching, reconstruction and validation are presented, where the global and local feature descriptors can be employed to effectively detect the features of point data of cattle, and the partitioned feature data can be iteratively matched and reconstructed into whole cattle. In-field experimentation results are presented to validate the measurement calibration.

**Figure 1:**
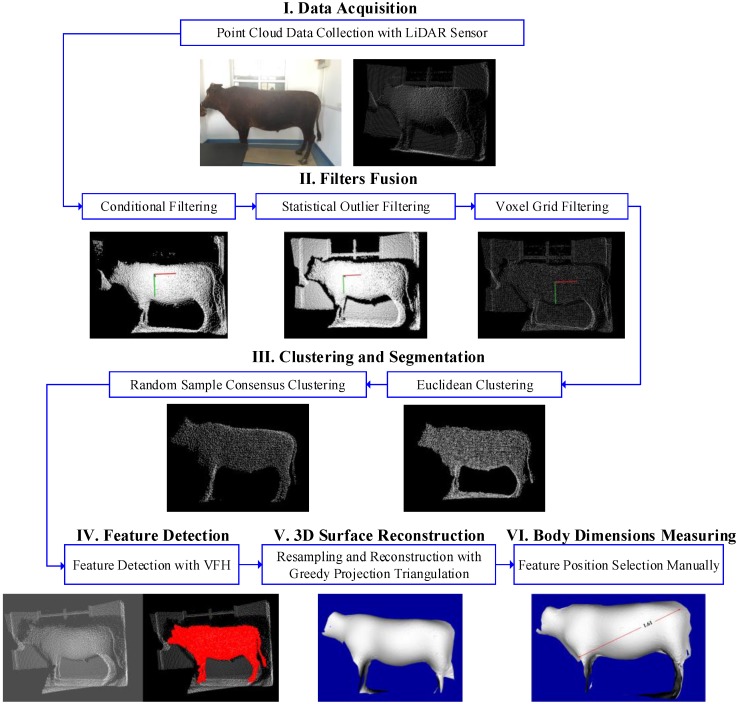
Flowchart of non-contact measurement of body dimensions for Qinchuan cattle with LiDAR sensor.

Figure 1 illustrates the scheme of the methodology proposed to measure the live Qinchuan cattle, Under the collection of PCD with an IFM O3D303 (IFM Inc., Essen, Germany) 3D LiDAR sensor, the filter fusion with conditional filtering, statistical outlier filtering and voxel grid filtering are fused to remove the noises. The segmentation with Euclidean clustering and RANSAC clustering are used to acquire the target cattle point cloud. With the feature detection a 3D surface reconstruction is used to obtain the 3D model of the Qinchuan cattle. Finally, the measurement results after calibration are obtained by selecting the positions of the body size in the 3D cattle model. All the trials and experiments, data processes and analysis are achieved with C++/C# programming language at Visual Studio with Point Cloud Library (PCL).

The rest of this paper is organized as follows: Section 2 describes the proposed method in detail. In Section 3, the experimental validation and corresponding discussions of the performance of body dimension measurement for the cattle are presented. The considerations, conclusions and future work are drawn in the last section.

## 2. Materials and Methods

### 2.1. Data Acquisition and Preprocessing

#### 2.1.1. Data Acquisition and Body Dimensions

**Figure 2:**
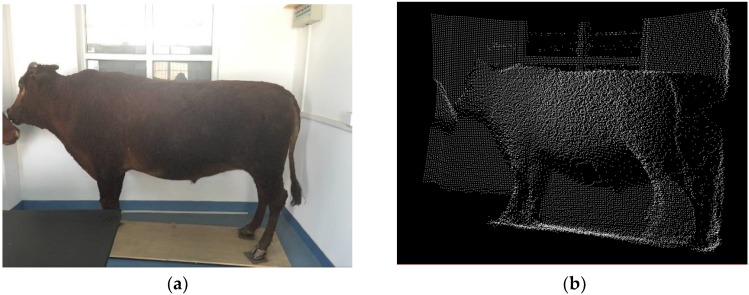
The 3D PCD acquisition for Qinchuan cattle, where the 3D image of LiDAR PCD shows the basic contour of target cattle: (**a**) Shown in RGB; (**b**) Shown in 3D image.

In this paper the O3D303 3D LiDAR camera is employed to collect the original PCD of cattle. It is a new type of depth camera with small volume and high frame rate, which can capture 3D information of targets in real time based on the principle of time-of-flight (ToF). It illuminates the scene with infrared light, and then calculates the distance between the camera and the nearest surfaces point by point with the unit of metric criterion. The sensor has the angle of aperture of 60° × 45° (horizontal × vertical) and its image resolution is 176 × 132 pixels. It has an Ethernet interface without any extra power supply, where the camera can be connected to Personal Computer (PC) via an Ethernet network cable. To capture the clear and complete target at an excellent view, the LiDAR sensor is supported on a common camera tripod. The 3D PCD acquisition for cattle with LiDAR sensor O3D303 is shown in Figure 2, where the 3D image after transformation shows the basic contour of cattle target.

**Figure 3:**
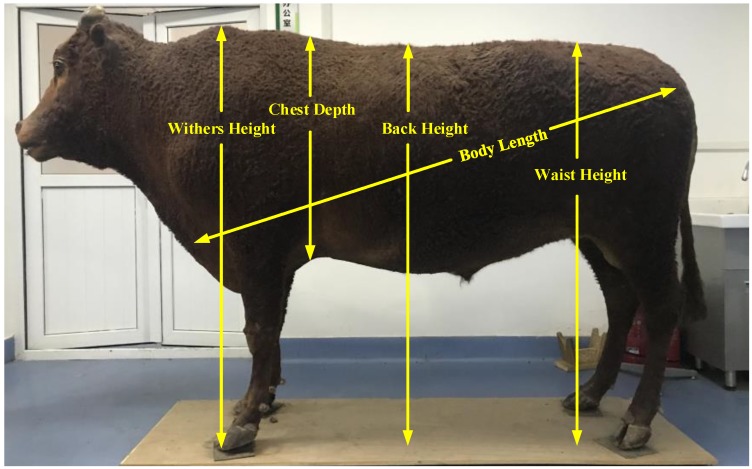
Scheme of five body dimensions of real specimen of adult Qinchuan cattle.

Five body dimensions of the Qinchuan cattle (withers height, chest depth, back height, body length and waist height) could be measured, respectively, as shown in Figure 3.

A schematic diagram of the measurement position of the body sizes is presented in the figure. The real live cattle specimens used were provided by the Ministry of Agriculture and Rural Affairs National Beef Cattle Improvement Center (Yangling, Shaanxi Province, China).

#### 2.1.2. Preprocessing with Filters Fusion

The filtering of PCD is the first and the most significant process of 3D point cloud preprocessing, which can drastically affect the subsequent processes and analysis like clustering segmentation, body feature detection, 3D surface reconstruction model and measurements. Due to the noise points caused by the sensor, different operations and the interferences of the external light source, and the discrete outliers produced by the backgrounds, the original 3D PCD needs to be preprocessed to remove the useless and irrelative PCD. The different filters of different purposes are fused to obtain the optimal filtering effects. With the considerations of noises cancelling, outliers’ removal and compressing of PCD, Conditional Removal Filter (CRF) Statistical Outlier Removal Filter (SORF) and Voxel Grid Filter (VGF) are fused to obtain the clear, complete and compressed target data.

**Figure 4:**
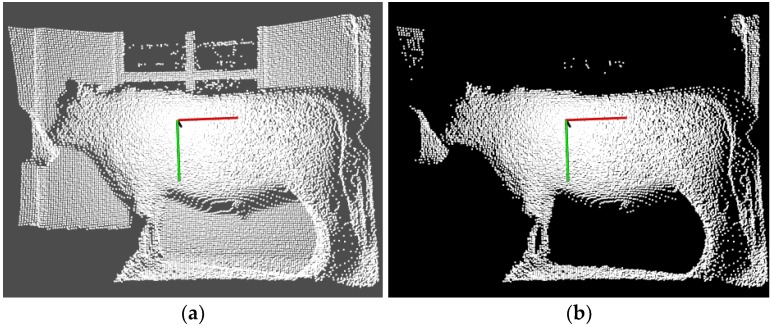
The filtering results of CRF, where many noises related to the distance are removed clearly: (**a**) Original PCD; (**b**) Filtering results with CRF.

Firstly, considering the background noises caused by the distance between the target cattle and the camera, the CRF is simple and feasible, and suitable to directly process the 3D coordinate position of the point within a certain threshold. Therefore, the simple CRF is employed to quickly remove the *Y*-axis data, and to partially save *X*-axis with the range of (−1.25, 1.00) and *Z*-axis of (1.50, 3.00). For the Qinchuan cattle is a large body animal, the clear and complete contour data of the cattle could be acquired only the distance that lies at the range of (1.50, 3.00). If the distance is beyond this range, most of the PCD could not reflect the whole contour of the cattle in this paper. The CRF filtering results are shown in Figure 4, where many noises related to the distance are removed clearly.

**Figure 5:**
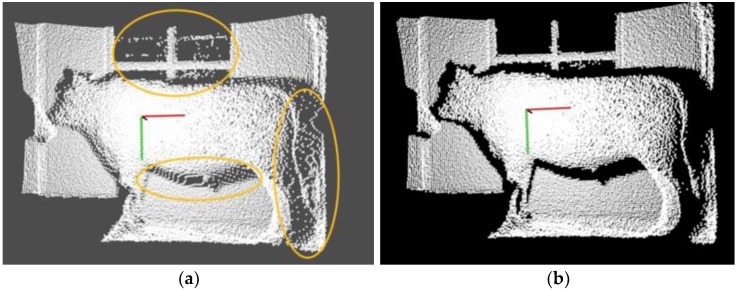
The filtering results of SORF, where partial outliers are removed and the whole contour of target are well preserved: (**a**) Original PCD; (**b**) Filtering results with SORF.

Secondly, to cancel sparse, discrete and useless outliers of the original data, which belong to Gaussian distribution and interfere with the target information processing to certain extent, the SORF is utilized on the principal of calculating the distances between the adjacent points of each point and setting the mean value and standard deviation of distances as thresholds. This filtering method is suitable for filtering common and obvious outliers. Figure 5 shows the results of this filtering with the thresholds setting shown in pseudo code of Algorithm 1, where the partial outliers are removed and the whole contour of target cattle are well preserved (outlier removal is expressed by yellow circles).

**Figure 6:**
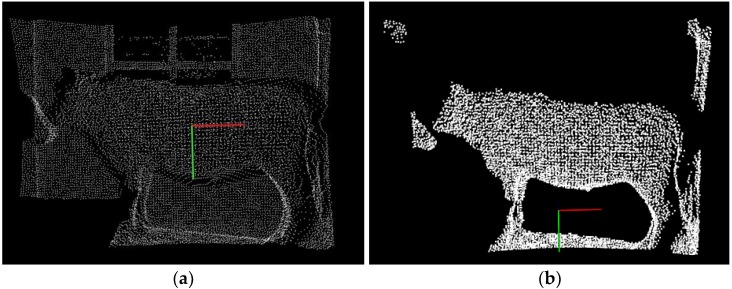
The filtering results: (**a**) Results of VGF, where many PCD are compressed and the basic contour of target are well preserved; (**b**) results with three filters fusion, where most noises and outliers are well removed, and the data is compressed with the target contour preserved.

Finally, to reduce subsequent computational complexity, the VGF [53] is employed to perform downward resampling without destroying the geometric shape of the PCD. With a 3D grid of the PCD created, just like a box-shaped 3D cube, called a leaf or voxel grid, which is determined by 3D coordinate variables, and the center of gravity of all points within each leaf is calculated, using the center of gravity as the sampling point to replace the other points in the leaf, and the data compression is realized. It is found that this filtering is a little time-costing instead of using the voxel center, and its filtering result could represent a relevant accurate surface with these sampling points. If the length and width of the voxel leaf is set as 3 cm, both the optimal compression and the geometrical shape preservation can be obtained. The filtering algorithm is as follows in Algorithm 1. Figure 6a shows the results of VGF, where many PCD are compressed at a ratio of over 30% and the basic contour of target cattle are well preserved. Figure 6b shows filtering results of three filters fusion, where most noises and outliers are well removed and the original whole shape of target is effectively preserved.

**Algorithm 1.** Filtering with three filters fusion**Input: ocloud**  % Original point cloud input data **Output: fcloud**  % Filtered point cloud output data 1. InputCloud ← *ocloud*   % Putting the original data into the filters container 2. Condition ← −1.25 < *x* < 1.0 && 1.5 < *z* < 3.0  % Setting the CRF filtering condition 3. KeepOrganized ← true  % Keeping the point cloud structure 4. *ccloud*← CrFilter(*ocloud*)    % Filtering with CRF 5. MeanK ← 60      % Setting the mean distances threshold of SORF as 60 6. StddevMulThresh ← 1    % Setting the outlier deviation threshold of SORF as 1 7. *scloud*←SorFilter(*ccloud*)  % Filtering with SORF 8. LeafSize ← (0.03 f, 0.03 f, 0.03 f) % Setting the grid of VGF as $3{\;\mathrm{cm}}^{2}$ 9. *fcloud*←VgFilter(*scloud*)    % Filtering with VGF

From the traits and advantages of three filters and their filtering effects above, only single CRF can cancel the useless point cloud dataset of a stationary specimen. In the indoor environment, the distance between the camera and the target is known, and only one or two filters could not sufficiently satisfy the preprocessing. In practice, live natural cattle are constantly moving, and all the point cloud dataset has an uneven distribution. To achieve a clear target contour and ensure the consistency of the filtering, the fused filtering of CRF, SORF and VGF is applied for real-time preprocessing.

### 2.2. Clustering Segmentation

#### 2.2.1. *K*-Means Clustering with *KD*-Trees Searching

After the preprocessing, there still exists some target adhesion data. To segment the irrelevant adhesions and to save the target data, the clustering segmentation is employed to obtain the clear and complete silhouette. For the spatial position and geometry shape of PCD, the *K*-means clustering using *kd*-trees searching [54] is firstly employed to segment the spatial-related data. Based on the distance relationship of adjacent points, this clustering method groups the points with similar Euclidean distance features into same clusters iteratively.

**Figure 7:**
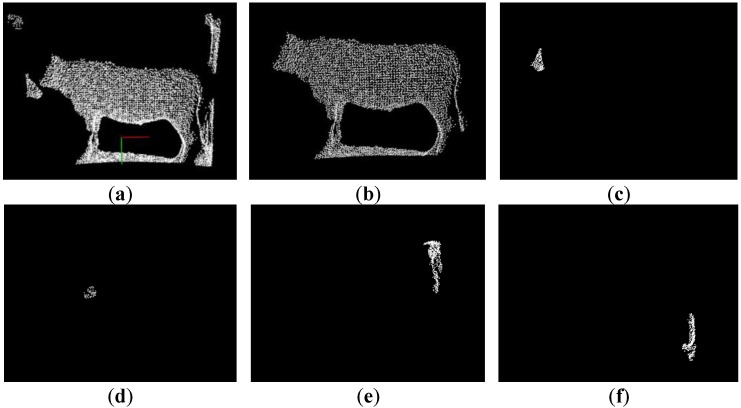
Each cluster segmented by *K*-means clustering: (**a**) Input preprocessed data; (**b**) Target cluster with all the body contour well preserved; (**c**–**f**) other cluster.

**Figure 8:**
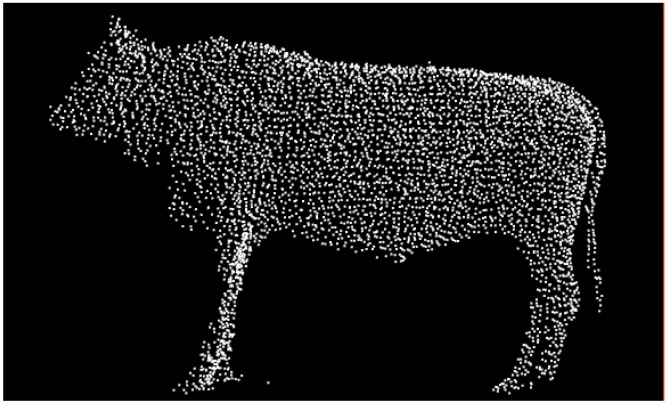
The segmentation results with RANSAC and its parameters set in Algorithm 2, where most of the background data adhered to target cattle at the bottom has been segmented.

Figure 7 illustrates each cluster segmented by K-means clustering using *kd*-trees searching, where all the isolated outliers and clusters of the input preprocessed data have been segmented. The biggest cluster is the target cluster shown in the Figure 8b, which contains the clear shape and the ground information adhered to the four legs of cattle at the bottom. Still, for the point data is scanned by the ToF sensor, there are few floating clusters separately isolated, such as the grassland, walls, stalls, other live cattle and others closely adherent to the target cattle. Therefore, the *K*-means clustering is not feasible to operate preprocessed point cloud directly, and it is necessary to resampling or to re-extract the other objects according to the practical target cattle situation.

#### 2.2.2. Plane Segmentation with RANSAC

After the segmentation of *K*-means clustering, the background adhered to the target cattle belongs to a same clustering, and it is necessary to segment the cattle from the ground for subsequent feature detection. The RANSAC algorithm [55] is based on a set of pre-segmented datasets containing abnormal data (i.e., adherent outliers or irregular objects), and estimates the mathematical model parameters of the data iteratively. The segmentation processing with *K*-means clustering and RANSAC is shown in Algorithm 2, and its corresponding results are shown in Figure 8. Compared to the segmentation of Figure 7b, Figure 8 shows that most of the background data adhered to target cattle at the bottom has been extracted.

**Algorithm 2.** Segmentation processing with *K*-means clustering and RANSAC**Input: fcloud**   % Input preprocessed point cloud data **Output: segcloud** % Segmented point cloud data 1. InputCloud ← *fcloud*     % Put the input data into the segmentation container 2. ClusterTolerance ← 0.05    % Set the cluster searching radius as 0.05 m 3. MinClusterSize ← 50       % Set the minimal clusters quantity as 50 4. *ecloud* ← EuExtract(*fcloud*)     % Segment input data with *K*-means clustering 5. ModelType ← SACMODEL_PLANE % Set the segmentation model type as planar model 6. MethodType ← SAC_RANSAC    % Get parameter estimation with RANSAC 7. DistanceThreshold ← 0.02        % Set the distances threshold in the model as 0.02 m 8. *segcloud* ← RANExtract(*ecloud*)      % Segment the point cloud data with RANSAC 

### 2.3. Feature Detection of FPFH

#### 2.3.1. FPFH Descriptor

After a series of clustering process at each practical scene, a large batch of PCD files are produced. It is an inaccurate and time-consuming task to manually classify target cloud cluster files, and it is necessary to find a feature descriptor to automatically detect the correct target point cloud clustering files. The point cloud feature descriptor is mainly used for describing the local or global geometrical and topological features of 3D PCD and involves all the characteristics of 3D PCD. The Viewpoint Feature Histogram (VFH), a global 3D point cloud feature descriptor, uses model feature of known feature model library to detect point cloud files. It derives from Fast Point Feature Histogram (FPFH) descriptors which have the ability to recognize spatial 3D objects, and adds the extra view variables which could maintain scaling invariance and distinguish different poses of 3D objects.

The feature component of FPFH, representing the 3D surface shape, computes the angles in the cluster between the connecting line between each point in the cluster and cluster centroid and the unit normal line of cluster surface, then count the angles information as a histogram. The viewpoint feature, different from the FPFH, is expressed to all angles as a histogram, where the cluster surface fitted by the least square method using *K*-neighborhood of each point in the cluster, and the unit normal vector of each point in this cluster surface computed, all the angles between the connection line between the centroid of this cluster and the viewpoint and this unit normal vector of each point in this surface are obtained.

**Figure 9:**
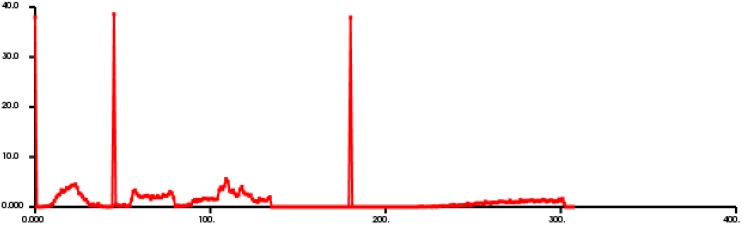
The FPFH of Figure 8, where the horizontal axis (unit: point numbers) represents each subinterval of VFH expressed by 308 float point in total, and the ordinate axis (unit: %) the feature estimate for each subinterval by percentage of the number of points in the subinterval and in the horizontal axis, the former 128 float points represent the viewpoint feature component while the latter 180 float points represent features of FPFH.

The segmented clusters are calculated with FPFH descriptor, and the feature curve of VFH is extracted shown in Figure 9, where the horizontal axis represents each subinterval of VFH expressed by 308 float point in total, and the ordinate axis the feature estimate for each subinterval by percentage of the number of points in the subinterval and in the horizontal axis, the former 128 float points represent the viewpoint feature component while the later 180 float points represent features of FPFH.

#### 2.3.2. Feature Models Library and Feature Matching

After the FPFH descriptor calculation extraction, the feature detection of PCD involves comparing the FPFH one by one with known cattle’s models to identify whether this cluster is the target point cloud cluster file. Therefore, it is significant to construct feature model library for Qinchuan cattle before the classifications of clusters. At first, a set of target cattle PCD of clear and well defined are selected, and then the *K*-means clustering and RANSAC algorithm are employed to segment the object’s point cloud cluster. After that, the cattle point cloud files from all the cluster files are manually extracted and their VFHs are calculated. Finally, all the FPFH files are saved and converted into the Fast Library for Approximate Nearest Neighbors (FLANN) data format [56]. A *kd*-trees index created by the FLANN data format, a fast disk-file searching structure, is saved in current directory in disk to reduce the computational capacity for the subsequent cluster recognition.

With the completion of the feature model library, the cluster classifier is created to automatically detect features from clustered files. The specific filtering preprocess, clustering segmentation and FPFHs calculation of clusters for all the original point cloud files captured on the spot are carried out. The FPFH file of each cluster to be matched is compared to every FPFH file in feature model library, and the similarity of two FPFH files, the Euclidean distance between two features of FPFH, are calculated one by one. All the similarities of each file in the model library are sorted, and the maximum similarity represents the successful matching. If the minimum similarity is smaller than the given threshold, then the FPFH cluster files to be matched should be deleted.

**Figure 10:**
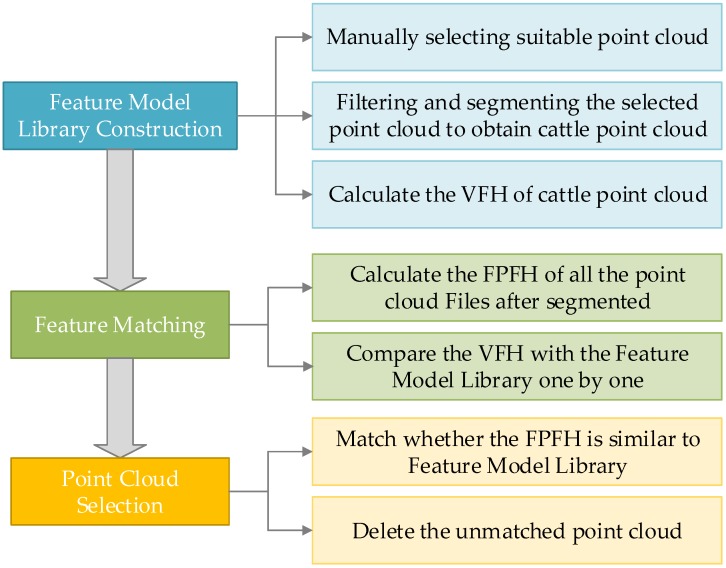
The feature matching process with FPFH descriptor which involves three steps: to construct the feature model library, to match the FPFH Feature, and to select the matched FPFH files.

**Figure 11:**
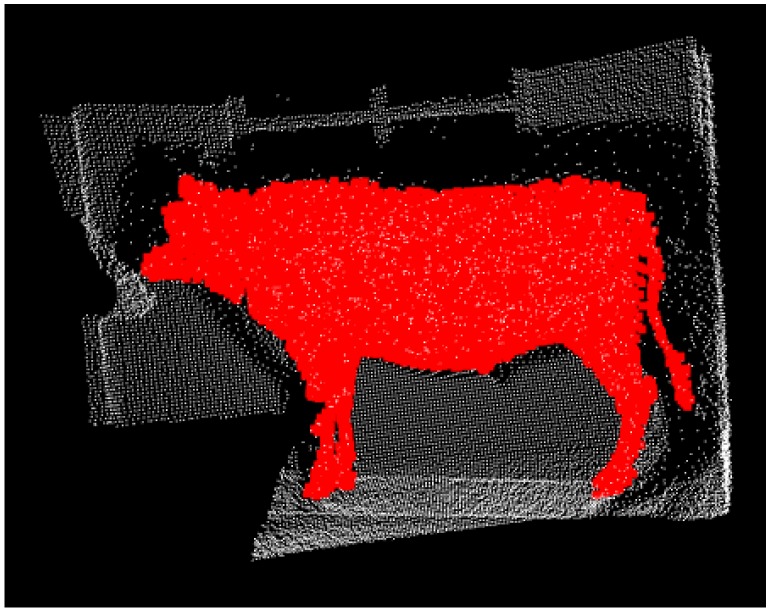
The matching result with FPVH descriptor of Figure 9, where the red portion points out the whole contour of cattle of Figure 2b.

Figure 10 shows the feature matching process with FPFH descriptors, which involves three steps: to construct the feature model library, to match the FPFH Feature, and to select the matched FPFH files:

(1) Construct the feature model library. With 3D PCD collection of some live cattle on the spot, the specific features of cattle are selected. Several typical groups of point clouds are filtered, clustered and segmented, and then, several groups of cattle point cloud manually are decided as known target feature cluster model. Finally, the FPFH feature descriptors of each cluster are computed to construct the training library of the feature model.
(2) Feature matching. The FPFH of all the point cloud files are extracted with a clustering classifier, and the input clustering files to be detected are compared with the feature model library one by one.
(3) Select point clouds. The Euclidean distance is calculated as a similarity index to match whether the FPFH of the point cloud is similar to the feature model library. If the distance is beyond the given threshold, which is called a mismatch, the feature cluster is removed by the classifier. The corresponding pseudo is shown in Algorithm 3. The matching result is shown in Figure 11 where the red portion indicates the whole contour of cattle.

**Algorithm 3.** Feature Detection with FPFH descriptor**Input: segcloud**   % Segmented point cloud data **Output: tcloud**     % Output target point cloud data 1. **initialize**
*n*, *VFH* % *n* representing the number of point cloud files after segmentation  % *VFH* representing the VFH of the Model Feature Library 2. **for**
*i* := 1, …, *n*
**do** 3.   NormalEstimation()  % Make a normal estimate 4.   VFH*i* ← calcVFH()   % Calculate the VFH of the point cloud 5.  **if** (VFH*i* – VFH) > thresh **then**  % Point cloud matching 6.    delete (pld)         % Delete the unmatched point cloud 7.   **end if** 8. **end for**

### 2.4. 3D Surface Reconstruction

#### 2.4.1. ICP Registration with BRKD-Trees Searching

After feature model library construction and the feature file extraction, the 3D surface of cattle is reconstructed to obtain a smooth and complete 3D surface data model for measuring. The reconstructed cattle surface enables an intuitive rendering of the scattered PCD for the subsequent body measurements. Due to the fact the 3D PCD is influenced by geometry of target cattle, the view scope of 3D camera and continuous movement of live cattle, it is necessary to stitch and register the different and scattered surface data into a whole and high-quality contour. The Iterative Closest Point (ICP) fast stitching and registration method [57,58] is utilized for the accurate registration. For an accurate and reliable method for registration of free form surfaces, the ICP algorithm is used for finding the rigid transformation iteratively between the target point set and the source point set so that two matching Euclidean distance satisfy the optimal match at a given convergence threshold.

Due to the construction difficulties of point cloud topology and geometry for the moving cattle, the parallel or bi-direction fast searching with the nearest neighborhood becomes the key issue. The random kd-trees searching [59] suits for the complex key point set matching at a high-dimension searching space. Due to the many iterative loop calculations, the BRKD-trees (Bi-direction Random *kd*-trees) searching method [60] is employed to better the efficiency of ICP registration algorithm. At the searching nearest point, the BRKD-trees method accelerates the point pairs searching process. 

**Figure 12:**
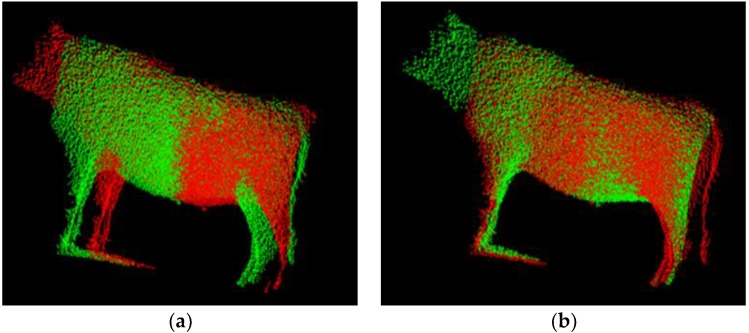
The ICP registration with BRKD-trees searching method of Figure 8: (**a**) Superimposed result of two partial point cloud series; (**b**) ICP registration result.

Figure 12 shows the ICP registration with BRKD-trees searching method, where the left image represents simple superimposed data of two partial point cloud series and the right image ICP registration result. The above comparison between superposition and registration points out that the superposition operations cause misshapen and even wrong cattle contours. However, in Figure 12, the ICP registration result also gives out many extra different point pairs which could result in superimposed surfaces to certain extent.

#### 2.4.2. Reconstruction with GPT

**Figure 13:**
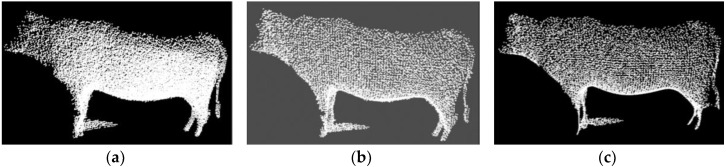
The entire comparison result: (**a**) Input registered data; (**b**) Filtered result with VGF; (**c**) Resampled result with MLS.

**Figure 14:**
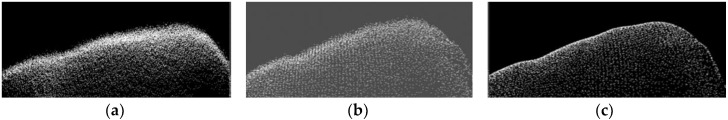
The local detail comparison result: (**a**) Input registered data; (**b**) Filtered result with VGF; (**c**) Resampled result with MLS.

Before surface reconstruction, there exists a need to smooth the modelled surface in case of region deviations. For the extra data after the registration, the VGF is employed to reduce the number of points to improve operational efficiency, and the Moving Least Squares (MLS) algorithm [61] to resample the registered data in case of overlapped surface. Based on high numerical accuracy and the approximating function of meshless method, the MLS is applied to fit cattle body curves and surfaces. Figure 13 and Figure 14 show the entire and local detail comparison results, respectively, where the number of point sets are better reduced, the geometry the contour is clear, smooth and well preserved.

**Figure 15:**
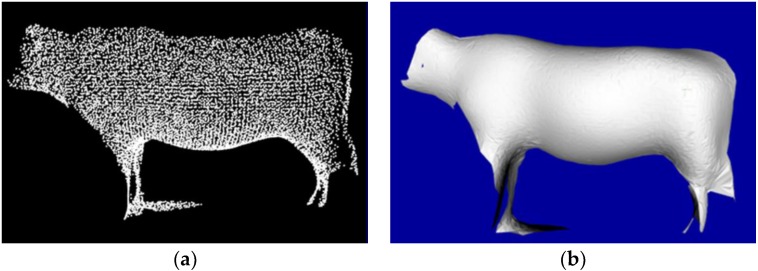
The result of surface reconstruction with GPT, where the reconstructed surface is smooth and has no holes: (**a**) Input Figure 13c; (**b**) 3D surface reconstruction model with GPT.

The Greedy Projection Triangulation (GPT) algorithm [62] is applied to reconstruct the cattle surface. Each point in 3D space and its surrounding K-neighborhood are projected into the tangent surface of the point for local Delaunay triangulation, so that the topological relation between the point and its surrounding points is obtained. Figure 15b shows the GPT reconstruction result of resampled data, where the high-quality reconstructed surface is smooth, and has no holes. By clicking the points on the reconstructed 3D surface model, the coordinates of marked points are obtained and the body dimensions of Qinchuan cattle are calculated.

### 2.5. Fitting Function

Because of the distance errors of the original measured object between the 3D surface model reconstructed based on the principle ToF sensor and its real dimensions, there is a necessity to construct a fitting model to revise the cattle dimension measurements. With the 3D PCD capture of different sizes of indoor spheres and cuboids by the ToF sensor, after a series of filtering, segmentation, clustering and recognition processes, the object dimensions of different photographic distances are obtained, where the dimensions data are fitted to the actual sizes. The surface fitting function and the curve correction function are presented in our previous work, respectively [52].

After the surface model reconstruction and according to the fitting model of ToF sensor, the feature measurement points can be selected to calculate the body dimensions of Qinchuan cattle. Using mouse events in the callback function of PCL for user interaction realization, the measurement points of body dimensions are feasibly calculated and automatically calibrated. According to the measured points in the feature parts of cattle selected manually, the real body dimensions are calculated by the projection distance of these selected points.

## 3. Experiments and Discussion

### 3.1. Experiments

**Figure 16:**
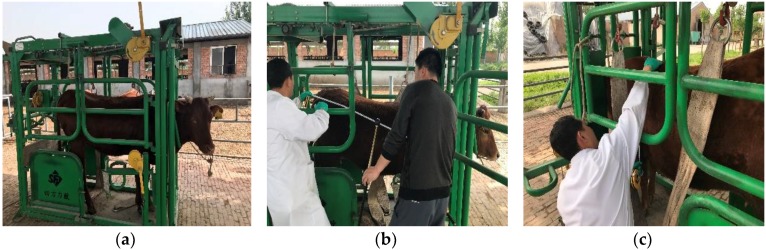
Manual measurement process of three cattle, which are kept in hold frame in case of cattle’s stress reactions: (**a**) Scenario of adult Qinchuan cow with ear mask of Q0159 settled in hold frame for measuring; (**b**) Scenario of body length measuring; (**c**) Scenario of withers height measuring.

According to the flowchart shown in Figure 1 and the methodology proposed above, the on-line and manual measurement for three live Qinchuan cattle are compared to validate the feasibility of the non-contact measurement method. The Qinchuan cattle used in the experiment become from the Ministry of Agriculture and Rural Affairs National Beef Cattle Improvement Center. The ear marks of the three adult Qinchuan cattle were Q0521, Q0145 and Q0159, respectively. To ensure the measurement accuracy at the manual measurement process, the cattle must be kept into the hold frame to hold the position of cattle definitely in case of cattle’s stress reactions. The manual measurement scenes of the three cattle are shown in Figure 16.

**Figure 17:**
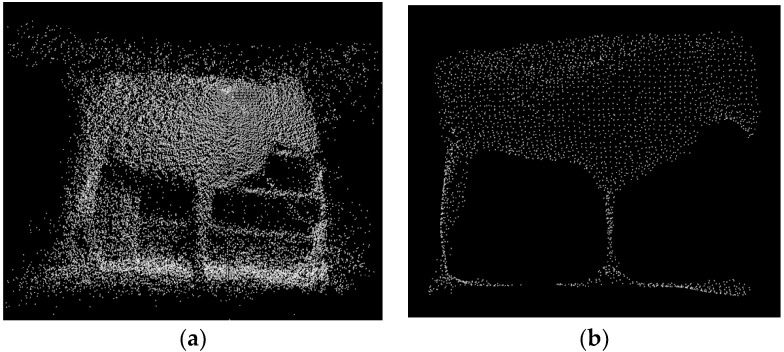
The illustration of original point cloud acquisition and preprocessed result of Q0159 cattle: (**a**) Original data; (**b**) Preprocessed result.

**Figure 18:**
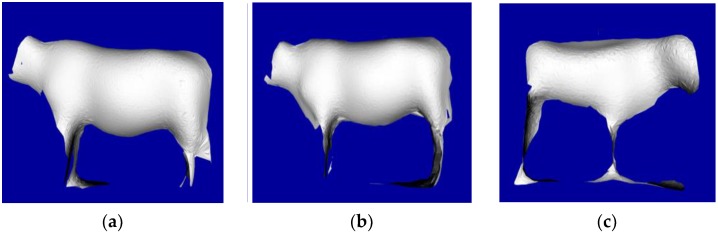
3D surface reconstruction results of three adult Qinchuan cattle: (**a**) Ear mask of Q0521; (**b**) Ear mask of Q0145 (**c**) Ear mask of Q0159.

After the manual measuring, the IFM O3D303 3D camera is employed to capture original PCD, and the original data and preprocessing result are illustrated in Figure 17. The surface reconstructions of three cattle after a series of filtering, clustering, segmentation, recognition, fitting and smoothing processing steps are shown in Figure 18. After the manual selection of the measurement points of the feature parts, the five body dimensions are calculated.

### 3.2. Discussion

**Figure 19:**
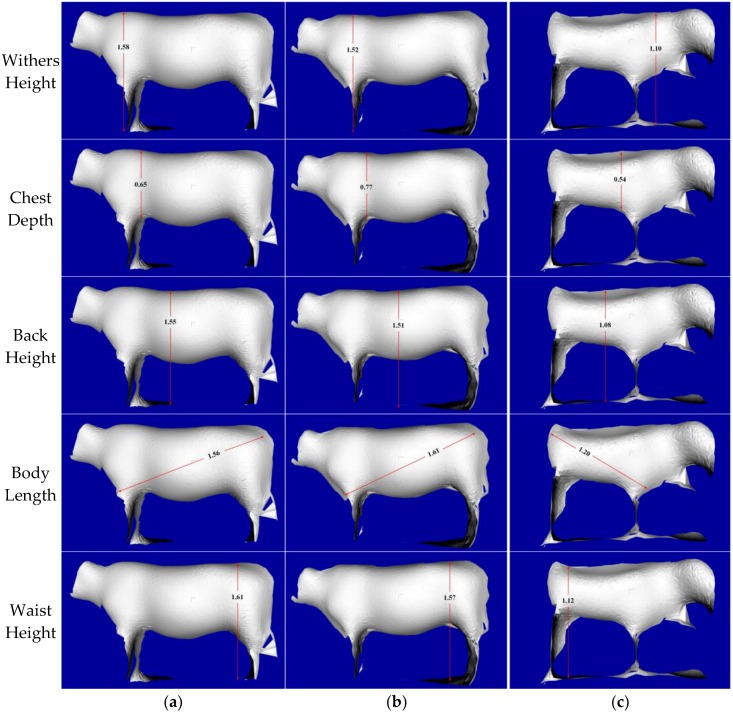
Non-contact measurement results of five body dimensions for three live Qinchuan cattle: (**a**) Q0521 steer at a distance of 1.57047 m; (**b**) Q0145 steer at a distance of 1.78572 m; (**c**) Q0159 steer at a distance of 1.54938 m.

With the calculation of specific feature parts of cattle and automatic correction function of 3D camera, the non-contact measurement results of five body dimensions for three live Qinchuan cattle are shown in Figure 19, where the corresponding photographic distances are computed.

Each steer’s manually measured values and the corresponding measurement deviations are presented in Table 1, Table 2 and Table 3 respectively, where the initial measurement value represents the direct project calculation without any fitting and correction function, and the corrected or final measurement value is the final measuring data after fitting and corrections; the Initial Deviation represents the error between the initial measurement value and manual measuring value, and the correction deviation is the final error between the final measured value and manually measured value.

From the three tables, for the five body dimension parameters, the maximum final deviation is equal to 2%, where the waist height is shown in Table 3; and the minimum final deviation is close to 0.2%, where the back height is listed in Table 1. The measuring and deviation values in Table 1 and Table 2 show that two cattle have similar size and their data distributions are close. However, the physique data in Table 3 is smaller, and the back line is not clear as the bigger one like Q0521 or Q0145, where the manual selection of body height, back height and waist height likely suffered a few deviations. For the practical manual accurate measurement of adult cattle, five to eight millimeter deviations are common. Nevertheless, the error of about 2 mm, within 2% or so, which could be acceptable and greatly improve the efficiency of measurement in the case of any stress response.

For the future, except for the fitting and correction parameter that are obtained alone in Matlab and applied to this methodology, the non-contact measurement of body dimensions is performed in Visual Studio 2016 with PCL 1.8 (CPU Intel i7 3.4G-Eight cores, Gloway DDR4 RAM of 64G, Windows 10 of 64-bit, Nvidia GeForce GTX1060 of 8G). The running time of all the C# code is less than five seconds from the acquisition, filtering and clustering segmentation to reconstruction. The manual selection operation of the feature part could cost close to four minutes for enlarging each feature part to obtain the precise points. In brief, the cost time of whole non-contact measurement of one adult Qinchuan steer could be five minutes. Compared with about 30 to 70 min required to precisely measure one adult steer manually, it could save much more time and greatly reduce the required labor.

## 4. Conclusions

In this paper, a novel approach of non-contact measurement for Qinchuan cattle body dimensions with a 3D ToF camera is proposed. After PCD is captured by a 3D sensor, and a series of filter fusion, K-means clustering with *kd*-tree searching, plane segmentation of RANSAC, feature detection of FPFH, ICP registration of BRKD-tree searching and reconstruction of GPT processing steps, combined with surface fitting function and curve correction function, five body dimension parameters are computed, which involve withers height, chest depth, back height, body length and waist height. Taking the manual precise measurement data as validation criterion, this methodology is verified with three live cattle and the experimental results show that the final deviations are close to 2 mm and within about 2% and thus meet the demands of time cost and accuracy.

This approach has verified the feasibility of non-contact measurement of for adult large physique animals, and it will greatly improve the development of healthy growth, animal welfare, automated precision feeding, animal quality improvement and genetic breeding. However, due to the different sizes ranging from calves to adult cattle, it is necessary to construct different measuring systems for their body dimensions. In the experiments performed in the field, it was difficult to obtain the whole silhouette several times due to the continuous movement of this breed. We often obtain the PCD of the partial body at one time, where it could lead to the wrong registration and reconstruction. For this problem, in the near future we will continue local optimization for fast registration, and explore the feasibility of using a transfer learning algorithm for transferring partial body features and a learning structure to achieve automatic sensing feature point regardless of the physique size of large animals.

## Figures and Tables

**Table 1:** Measuring values of animal Q0521 at the photographic distance of 1.57047 m. (unit: m)

Ear Mask Q0521 Cattle	Withers Height	Chest Depth	Back Height	Body Length	Waist Height
Manual Measuring Value (m)	1.56900	0.65300	1.55600	1.59100	1.59800
Initial Measuring Value (m)	1.41508	0.57622	1.38628	1.39373	1.43797
Corrected/Final Measuring Value (m)	1.58461	0.64525	1.55236	1.56071	1.61025
Initial Deviation	9.81%	11.76%	10.91%	12.40%	10.01%
Correction/Final Deviation	1.00%	1.19%	0.23%	1.90%	0.77%

**Table 2:** Measuring values of animal Q0145 at the photographic distance of 1.78572 m. (unit: m)

Ear Mask Q0145 Cattle	Withers Height	Chest Depth	Back Height	Body Length	Waist Height
Manual Measuring Value (m)	1.53400	0.75800	1.51600	1.58400	1.55800
Initial Measuring Value (m)	1.27672	0.64814	1.26818	1.35469	1.31889
Corrected/Final Measuring Value (m)	1.52120	0.77225	1.51103	1.61410	1.57145
Initial Deviation	16.77%	14.49%	16.35%	14.48%	15.35%
Correction/Final Deviation	0.83%	1.88%	0.33%	1.90%	0.86%

**Table 3:** Measuring values of animal Q0159 at the photographic distance of 1.54938 m. (unit: m)

Ear Mask Q0159 Cattle	Withers Height	Chest Depth	Back Height	Body Length	Waist Height
Manual Measuring Value (m)	1.12200	0.54100	1.10100	1.19600	1.14300
Initial Measuring Value (m)	0.98878	0.48211	0.97186	1.07749	1.00453
Corrected/Final Measuring Value (m)	1.10256	0.53759	1.08370	1.20149	1.12013
Initial Deviation	11.87%	10.89%	11.73%	9.91%	12.11%
Correction/Final Deviation	1.73%	0.63%	1.57%	0.46%	2.00%

## Abbreviations

The following abbreviations are used in this manuscript:

LiDAR	Light Detection And Ranging
RANSAC	RANdom SAmple Consensus algorithm
VFH	Viewpoint Feature Histogram
FPFH	Fast Point Feature Histogram
ICP	Iterative Closest Point matching algorithm
PCD	Point Cloud Data
3D	Three Dimensional
MLS	Moving Least Squares resampling algorithm
ToF	Time of Flight
PC	Personal Computer
BDRKD-trees	Bi-direction Random K-D Trees searching method
MPFH	Model Point Feature Histogram
FPFH	Fast Point Feature Histograms
SAC-IA	SAmple Consensus based Initial Alignment algorithm
PCL	Point Cloud Library
CRF	Conditional Removal Filter
FLANN	Fast Library for Approximate Nearest Neighbors data format
SORF	Statistical Outlier Removal Filter
VGF	Voxel Grid Filter
BRKD-trees	Bi-direction Random *kd*-trees searching method
GPT	Greedy Projection Triangulation algorithm
